# Supplementary material for: Structural insights into E1 recognition and the ubiquitin-conjugating activity of the E2 enzyme Cdc34
Source: Nat Commun. 2019 Jul 24;10:3296. doi: 10.1038/s41467-019-11061-8 (PMC6656757; doi:10.1038/s41467-019-11061-8)
Supplement: Supplementary file 1 — Supplementary Information [file 41467_2019_11061_MOESM1_ESM.pdf]

Structural insights into E1 recognition and the ubiquitin-conjugating activity of the E2 enzyme Cdc34

Williams et al.

Supplementary Information Inventory:

Supplementary Tables 1 and 2

Supplementary Figures 1-7

Supplementary Table 1| Crystallographic Data and Refinement Statistics

	ScUba1-Cdc34$^{\Delta dist}$/Ub(a) complex	ScCdc34$^{\Delta dist}$ Apo	hCdc34B$^{\Delta dist}$ -Ub thioester mimetic
PDB ID	6NYA	6NYD	6NYO
Source	APS 24 IDE	APS 22 ID	APS 24 IDE
Wavelength (Å)	0.979	1.00	0.979
Resolution Limits (Å)	160.6-2.07 (2.10-2.07)	50-1.65 (1.71-1.65)	50-1.5 (1.55-1.50)
Space Group	$P2_1$	$P2_12_12_1$	$P2_12_12_1$
Unit Cell (Å) *a*, *b*, *c*	126.4, 68.5, 171.7	40.1, 49.0, 103.7	44.6, 55.7, 119.8
Unit Cell (°) α, β, γ	90, 110.7, 90	90, 90, 90	90, 90, 90
Number of observations	756228	166028	274843
Number of reflections	167880 (7183)	25422 (2513)	48439 (4739)
Completeness (%)	99.2 (86.4)	99.8 (100)	99.9 (99.7)
Mean $I/\sigma I$	10.6 (1.0)	20.0 (1.2)	17.5 (1.5)
$CC_{1/2}$	0.995(0.347)	0.981 (0.609)	0.997 (0.582)
R_{merge} [a]	0.135 (1.42)	0.089 (1.15)	0.069 (0.976)
R_{pim}	0.072 (0.781)	0.039 (0.511)	0.032 (0.464)
Refinement Statistics			
Resolution Limits (Å)	117.0-2.07 (2.12-2.07)	37.4-1.65 (1.71-1.65)	41.8-1.50 (1.54-1.50)
# of reflections (work/free)	165596/2005	23354/1999	46361/2000
Completeness (%)	99.0 (89.0)	99.7 (99.0)	99.8 (98.0)
Protein/solvent/ligand atoms	19573/909/223	1396/103/16	2154/319/46
R_{cryst} [b]	0.187 (0.303)	0.171 (0.283)	0.175 (0.258)
R_{free} (2000 reflections)	0.217 (0.330)	0.199 (0.319)	0.202 (0.262)
Bonds (Å)/ Angles (°)	0.003/0.600	0.01/1.07	0.006/0.786
B-factors: protein/solvent/ligand (Å^2)	48.3/40.0/62.1	43.7/50.7/68.9	23.8/33.9/33.9
Ramachandran plot statistics (%)			
favored	97.0	99.4	99.6
allowed	3.1	0.6	0.4
outliers	0.2	0	0
MolProbity score	1.37- 99th percentile (N=11650, 2.07 Å ± 0.25Å)	1.28- 97th percentile (N=7252, 1.65 Å ± 0.25Å)	1.06- 99th percentile (N=4775, 1.50 Å ± 0.25Å)

Parentheses indicate statistics for the high-resolution data bin for x-ray data.
a. $R_{merge} = \sum hkl \sum i |I(hkl)i - \langle I(hkl) \rangle| / \sum hkl \sum i \langle I(hkl)i \rangle$.
b. $R_{cryst} = \sum hkl |Fo(hkl)-Fc(hkl)| / \sum hkl |Fo(hkl)|$, where *Fo* and *Fc* are observed and calculated structure factors, respectively.

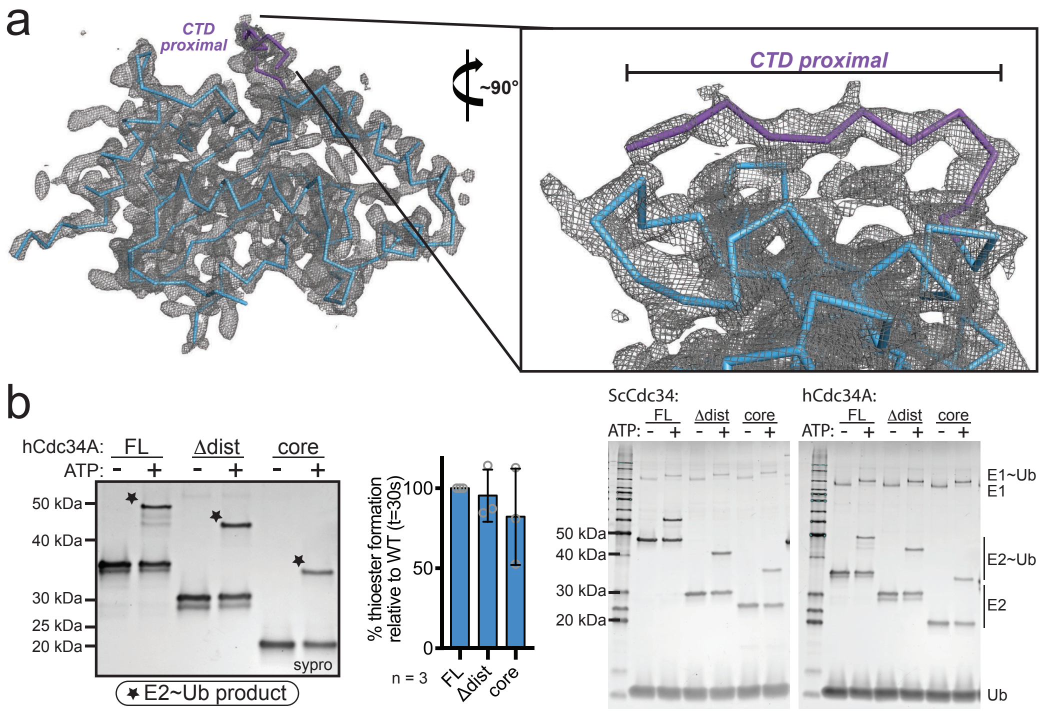

Supplementary Figure 1| E1-bound Cdc34 electron density maps and thioester transfer assays for truncation mutants

a, *left*, 2Fo-Fc electron density maps contoured at 1.0σ for Cdc34$^{\Delta dist}$ in the Uba1-Cdc34 structure with Cα-trace shown. *right*, zoomed in view of the ordered CTD^{prox} region. **b,** *left*, hCdc34A truncated mutant E1-E2 thioester transfer assay with representative image and quantification shown as in Fig. 1a, with three independent replicates. *right*, full gels for *S. cerevisiae* in Fig. 1a and human Cdc34 variant E1-E2 thioester transfer assays. Protein bands are labeled to the right of the images. Source data are provided as a Source Data file.

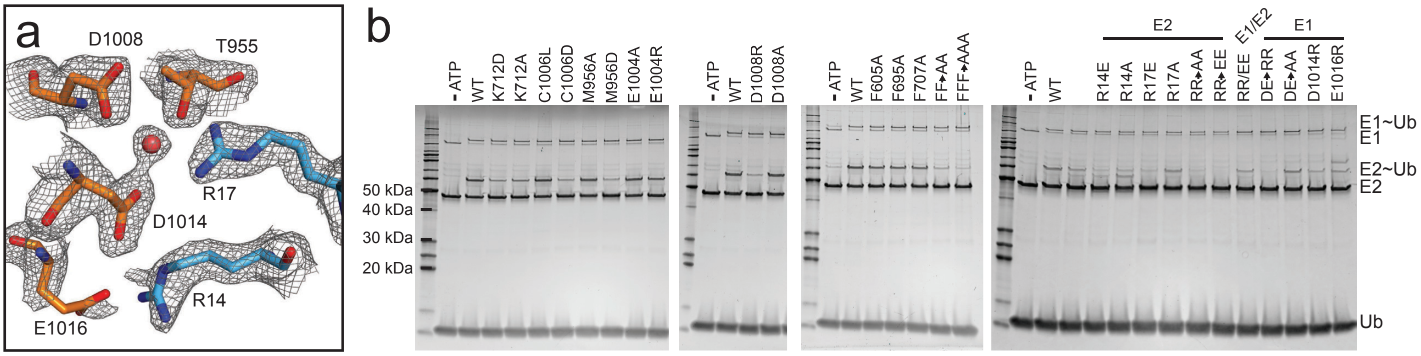

Supplementary Figure 2| Structure function analysis and representative electron density maps for Uba1/Cdc34 interfaces

a, 2Fo-Fc electron density maps contoured at 1.0σ for indicated residues and water at the Cdc34/UFD interface. **b,** Full gels for E1-E2 thioester transfer assays in Fig. 2.

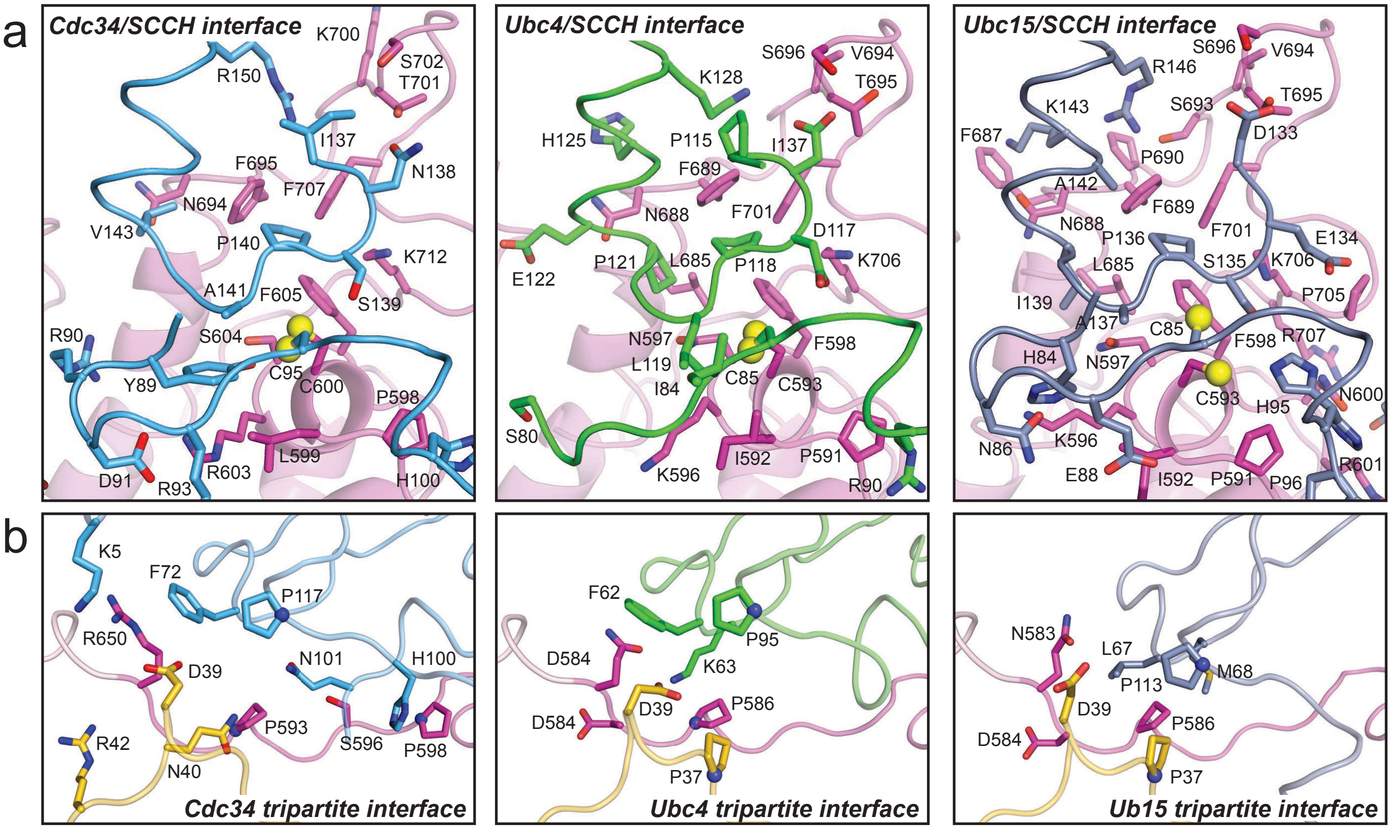

Supplementary Figure 3| Comparison of Cdc34, Ubc4, and Ubc15 SCCH and tripartite interfaces

a, Interaction networks between Uba1 SCCH and Cdc34 (*left*), Ubc4 (*middle*), and Ubc15 (*right*) represented as in Fig. 2. **b,** Interaction networks between Uba1 crossover loop, Ub(a), and Cdc34 (*left*), Ubc4 (*middle*), and Ubc15 (*right*) represented as in Fig. 2.

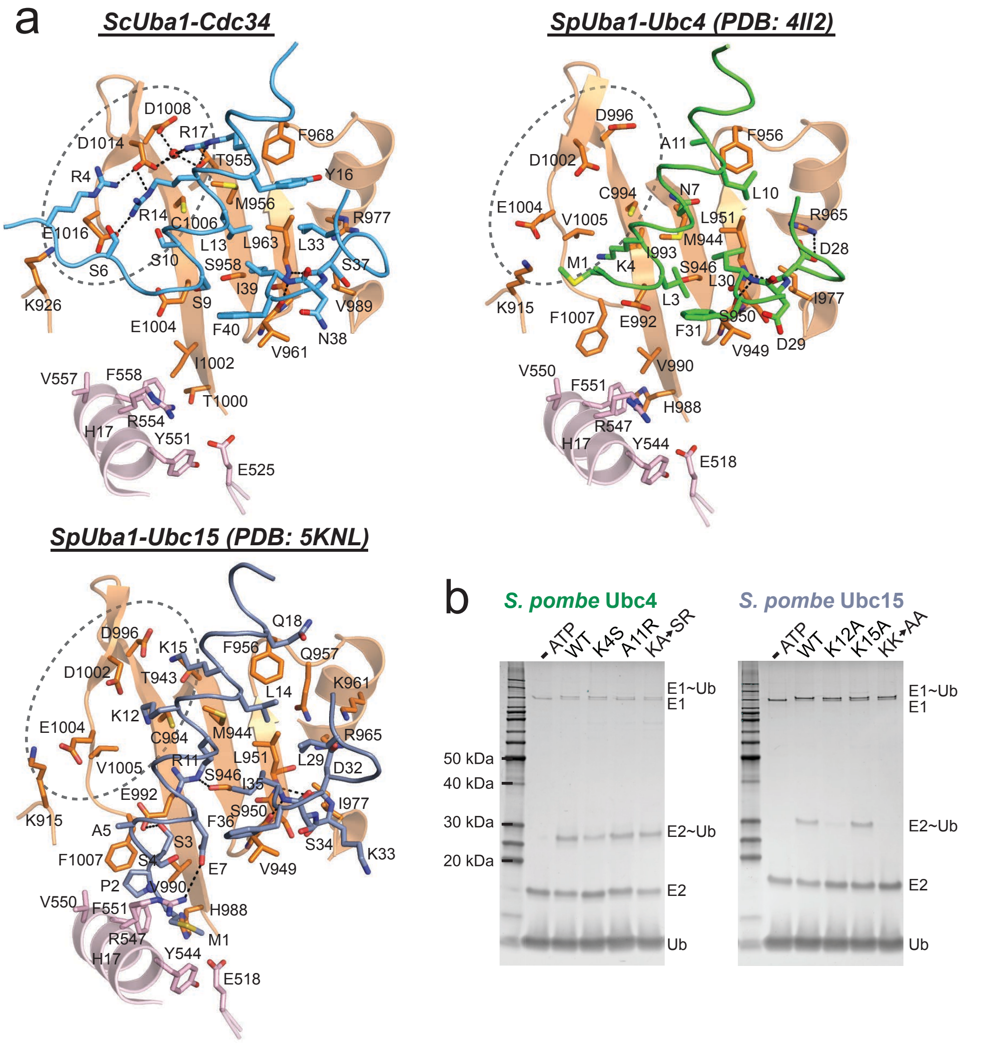

Supplementary Figure 4| Detailed interaction networks for UFD/E2 interfaces and corresponding E1-E2 thioester transfer assays

a, Detailed interaction maps corresponding to Fig. 4a. **b,** Full gels for E1-E2 thioester transfer assay of mutants for Ubc4 (*left*) and Ubc15 (*right*) as shown in Fig. 4b.

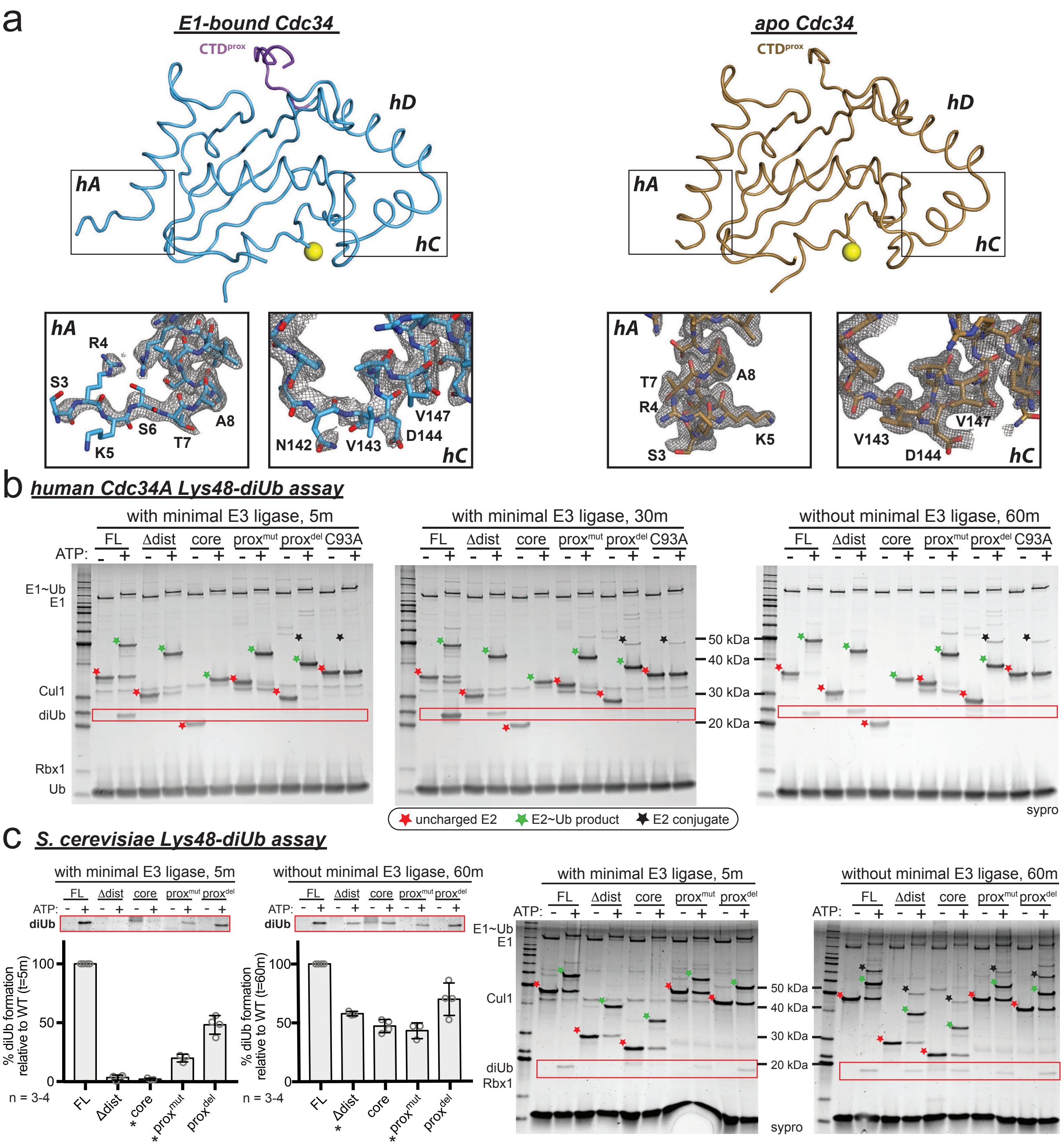

Supplementary Figure 5| apo and E1-bound Cdc34 structures and structure function analysis of CTD^{prox} mutants for human and yeast Cdc34

a, Crystal structures of *S. cerevisiae* E1-bound (*left*) and apo (*right*) Cdc34$^{\Delta dist}$ with regions of conformational change boxed and 2Fo-Fc electron density maps contoured at 1.0σ for these regions below. **b,** Full gels of K48-diUb assay in Fig. 5d for global hCdc34A mutants with minimal E3 ligase for 5m (*left*) and 30m (*middle*), and without E3 minimal ligase for 60m (*right*). **c,** Corresponding yeast Cdc34 mutants for Fig. 5d K48-diUb assay with and without minimal E3 ligase. *left*, data are represented by mean ± SD with individual replicates shown as gray circles and representative diUb bands labeled above. *right*, representative replicates are labeled and annotated with stars for clarity as in **b**. diUb product is indicated by a label and red box. Source data are provided as a Source Data file.

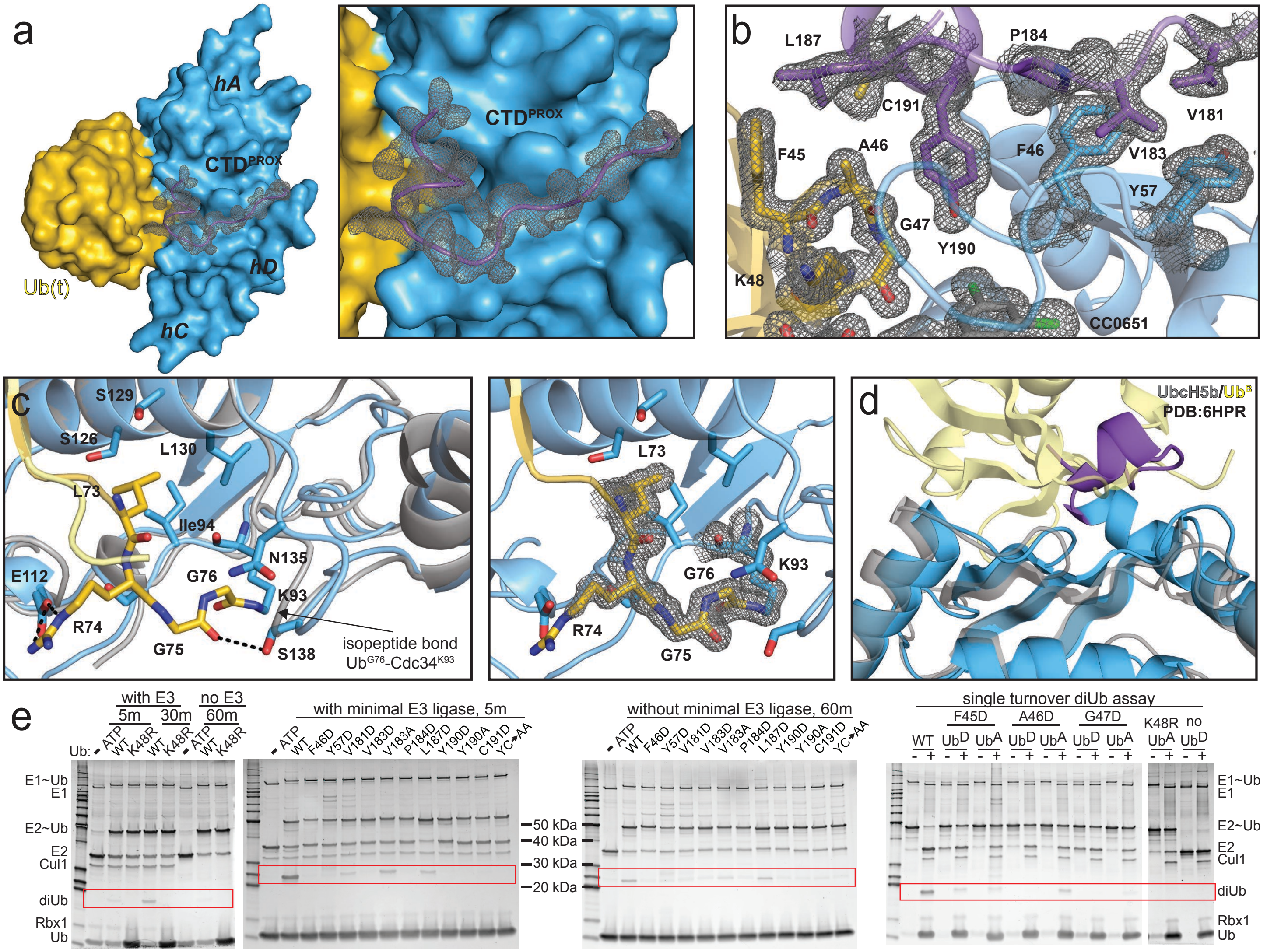

Supplementary Figure 6| Structure function analysis and electron density maps for hCdc34B$^{\Delta dist}$-Ub

a, Overall (*left*) and zoomed in (*right*) views of hCdc34B$^{\Delta dist}$-Ub structure as in Fig. 6A with 2Fo-Fc electron density maps contoured at 1.0σ for CTD^{prox}. **b,** Zoomed in view of CTD^{prox} contacts to the Ubc core and Ub(t) with 2Fo-Fc electron density maps contoured at 1.0σ. **c,** *left*, comparison of the network of contacts for the Ub(t) C-terminus in the hCdc34B$^{\Delta dist}$-Ub structure and hCdc34A/Ub (PDB: 4MDK). Specifically, our Cdc34-Ub structure reveals several contacts between the diglycine motif and Cdc34 Asn135/Ser138. Further, Ub Leu73 contacts Cdc34 hB, and Arg74 contacts several Cdc34 acidic loop residues. Additionally, Cdc34 active site residues Lys93, Ile94, and Ser95 contact the backbones of Ub Gly75, Leu73, and Arg74, respectively. *right*, 2Fo-Fc electron density maps contoured at 1.0σ for the Ub(t) C-terminus and isopeptide bond with Cdc34 C93K. **d,** Comparison of CTD^{prox} and noncovalent Ub (PDB: 6HPR) binding to the 'backside' of the Ubc core where backside Ub is yellow and UbcH5B is gray. **e,** *left*, full gels for Ub K48R control of K48-diUb assay. Source data are provided as a Source Data file. Full gels for multiturnover (*middle*) and single turnover (*right*) K48-diUb assays in Fig. 6c.

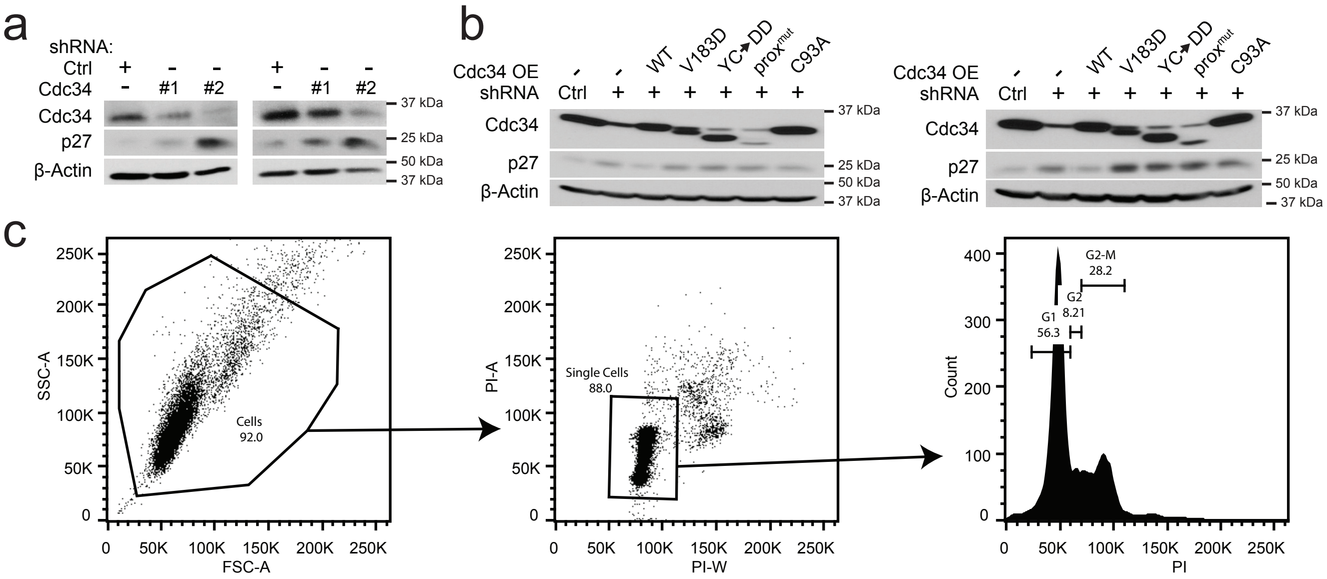

Supplementary Figure 7| Replicate Western blots and gating strategy for U2OS cell experiments

a,b, Additional replicate western blots of U2OS cells for Cdc34 and p27 after Cdc34 shRNA KD (a) and subsequent overpression of WT or mutant Cdc34 (b). **c,** Representative gating strategy for flow cytometry experiments to select for live single cells.

Supplementary Table 2| All PCR primers used in these studies

ScCdc34 – truncation at 170 w XhoI (use with a608)

K028 GGCGGCCTCGAGTTAATCTTGTTTCGATCTTTCCACTTC

hCdc34B – amplification from 1-202 (NcoI/XhoI)

K031 CGAAGCCATGGCCCAGCAGCAGATGAC
K032 CGAAGCTCGAGTTAGCTGTTGTCATTGGAAGGCACT

hCdc34A – stop codon at A176 to make core (1-175)

K033 ACCAAGGTGGACTAGGAGCGTGACGGCGTGAA
K034 TTCACGCCGTCACGCTCCTAGTCCACCTTGGT

hCdc34B – truncation via stop codon at Ser203

K029 GCCTTCCAATGACAACAGCTAAGATTTGCTTTACGACGAC
K030 GTCGTCGTAAAGCAAATCTTAGCTGTTGTCATTGGAAGGC

Amplifications of core (1-175) Ube2r2 for PET29NTEV (NcoI/NotI)
use with k424.

k426 GAAGAAGCGGCCGCCTATTCGGCCTTAGTGGCTGAAAC

Amplification of FL hCdc34B for PET29NTEV (NcoI/NotI)

k424 GCGCGGCCATGGCCCAGCAGCAGATGAC
k425 GAAAGCGGCCGCCTACGACTCCTCATTCCCAGAATC

ScUba1 mutagenesis primers

M956A (atg → gcg)

K223 GGGTTAGAAATAACAGCGCTTTCTTATGGCGTTTCTCTTC
K224 GAAGAGAAACGCCATAAGAAAGCGCTGTTATTTCTAACCC

M956D (atg → gac)

k384 GGGTTAGAAATAACAGACCTTTCTTATGGCGTTTCTCTTC
k385 GAAGAGAAACGCCATAAGAAAGGTCTGTTATTTCTAACCC

ScUba1 E1004A (gaa → gca)

ja143 GTATCTACAATGATTCTCGCAATTTGCGCAGATGACAAGG
ja144 CCTTGTCATCTGCGCAAATTGCGAGAATCATTGTAGATAC

ScUba1 E1004R (gaa → aga)

ja145 GTATCTACAATGATTCTCAGAATTTGCGCAGATGACAAGG
ja146 CCTTGTCATCTGCGCAAATTCTGAGAATCATTGTAGATAC

C1006L (tgc → ctc)

K225 CTACAATGATTCTCGAAATTCTCGCAGATGACAAGGAAGG
K226 CCTTCCTTGTCATCTGCGAGAATTTCGAGAATCATTGTAG

C1006D (tgc → gac)

k396 CTACAATGATTCTCGAAATTGACGCAGATGACAAGGAAGG
k397 CCTTCCTTGTCATCTGCGTCAATTTCGAGAATCATTGTAG

D1008A (gat → gct)

M1 GATTCTCGAAATTTGCGCAGCTGACAAGGAAGGAGAGGAT
M2 GATTCTCGAAATTTGCGCACGTGACAAGGAAGGAGAGGAT

D1008R (gat → cgt)

M3 GATTCTCGAAATTTGCGCACGTGACAAGGAAGGAGAGGAT
M4 ATCCTCTCCTTCCTTGTCACGTGCGCAAATTTCGAGAATC

F605A

k427 CATTGTGTACCCTACGTTCTGCCCCAAACAAGATTGATC
k428 GATCAATCTTGTTTGGGGCAGAACGTAGGGTACACAATG

F695A

k429 GTTACTGTTCAACGCCCCCAAGGATGCCAAGACTTC
k430 GAAGTCTTGGCATCCTTGGGGGCGTTGAACAGTAAC

F707A

k431 CCAACGGTGAACCAGCTTGGTCCGGTGCTAAG
k432 CTTAGCACCGGACCAAGCTGGTTCACCGTTGG

K712D (aag → gat)

K219 CATTTTGGTCCGGTGCTGATCGTGCTCCAACACC
K220 GGTGTTGGAGCACGATCAGCACCGGACCAAAATG

K712A (aag → gcg)

K221 CATTTTGGTCCGGTGCTGCGCGTGCTCCAACAC
K222 GTGTTGGAGCACGCGCAGCACCGGACCAAAATG

D1014R (gat → cgc)

K227 CAGATGACAAGGAAGGAGAGCGCGTTGAAGTTCCTTTC
K228 GAAAGGAACTTCAACGCGCTCTCCTTCCTTGTCATCTG

D1014A (gat → gcg)

K229 CAGATGACAAGGAAGGAGAGGCGGTTGAAGTTCCTTTC
K230 GAAAGGAACTTCAACCGCCTCTCCTTCCTTGTCATCTG

Supplementary Table 2| All PCR primers used in these studies

E1016R (gaa → cgc)
K231 GACAAGGAAGGAGAGGATGTTCGCGTTCCTTTCATTACC
K232 GGTAATGAAAGGAACGCGAACATCCTCTCCTTCCTTGTC

E1016A (gaa → gcg)
K233 GACAAGGAAGGAGAGGATGTTGCGGTTCCTTTCATTACC
K234 GGTAATGAAAGGAACCGCAACATCCTCTCCTTCCTTGTC

D1014R E1016R (stack onto D1014R)
K235 CAAGGAAGGAGAGCGCGTTCGCGTTCCTTTCATTAC
K236 GTAATGAAAGGAACGCGAACGCGCTCTCCTTCCTTG

D1014A E1016A (stack onto D1014A)
K237 CAAGGAAGGAGAGGCGGTTGCGGTTCCTTTCATTAC
K238 GTAATGAAAGGAACCGCAACCGCCTCTCCTTCCTTG

ScCdc34 mutagenesis primers

R14E (cgg → gag)
K239 GCACCGCTTCTAGCTTACTGTTAGAGCAATATAGAGAAC
K240 GTTCTCTATATTGCTCTAACAGTAAGCTAGAAGCGGTGC

R14A (cgg → gcg)
K241 GCACCGCTTCTAGCTTACTGTTAGCGCAATATAGAGAAC
K242 GTTCTCTATATTGCGCTAACAGTAAGCTAGAAGCGGTGC

R17E (aga → gaa)
K243 CTTACTGTTACGGCAATATGAAGAACTTACTGATCCTAAG
K244 CTTAGGATCAGTAAGTTCTTCATATTGCCGTAACAGTAAG

R17A (aga → gca)
K245 CTTACTGTTACGGCAATATGCAGAACTTACTGATCCTAAG
K246 CTTAGGATCAGTAAGTTCTGCATATTGCCGTAACAGTAAG

R14E R17E (cgg → gag, aga → gaa)
k317 GCACCGCTTCTAGCTTACTGTTAGAGCAATATGAAGAAC
k318 GTTCTTCATATTGCTCTAACAGTAAGCTAGAAGCGGTGC

R14A R17A (cgg → gcg, aga → gca)
k319 GCACCGCTTCTAGCTTACTGTTAGCGCAATATGCAGAAC
k320 GTTCTGCATATTGCGCTAACAGTAAGCTAGAAGCGGTGC

SpUbc4 K4S (aaa → agc)
K269 CATATGGCTTTGAGCAGAATTAACCGTGAATTAGCTGATC
K270 GATCAGCTAATTCACGGTTAATTCTGCTCAAAGCCATATG

SpUbc4 A11R (gct → cgt)
K271 GAATTAACCGTGAATTACGTGATCTTGGAAAAGACCCACC
K272 GGTGGGTCTTTTCCAAGATCACGTAATTCACGGTTAATTC
Stack A11R onto K4S for double mutant

SpUbc15 K12A (aaa → gcg)
K277 GCTAGTGAACAACTTCTTCGAGCGCAACTAAAGGAAATTC
K278 GAATTTCCTTTAGTTGCGCTCGAAGAAGTTGTTCACTAGC

SpUbc15 K15A (aag → gcg)
K279 CGAAAACAACTAGCGGAAATTCAAAAAAATCCTCCACAGG
K280 CCTGTGGAGGATTTTTTTGAATTTCCGCTAGTTGTTTTCG

SpUbc15 K12A K15A (aag → gcg) onto K12A
K297 CGAGCGCAACTAGCGGAAATTCAAAAAAATCCTCCACAGG
K298 CCTGTGGAGGATTTTTTTGAATTTCCGCTAGTTGCGCTCG

hCdc34A CTD^{prox} mutations

F46D (ttc → gac)
K063 GTGGCCATCGACGGGCCCCCAACACCTACTA
K064 TAGTAGGTGTTGGGGGCCCGTCGATGGCCAC

Y57D (tac → gac)
K065 CTACTACGAGGGCGGCGACTTCAAGGCGCG
K066 CGCGCCTTGAAGTCGCCGCCCTCGTAGTAG

V181D (gtg → gat)
K067 GAGCGTGACGGCGATAAGGTGCCCACCACG
K068 CGTGGTGGGCACCTTATCGCCGTCACGCTC

V183D (gtg → gat)
K069 GTGACGGCGTGAAGGATCCCACCACGCTGG
K070 CCAGCGTGGTGGGATCCTTCACGCCGTCAC

P184D (ccc → gac)
K071 GGCGTGAAGGTGGACACCACGCTGGCTA
K072 TAGCCAGCGTGGTGTCCACCTTCACGCC

Supplementary Table 2| All PCR primers used in these studies

Y190D (tac → gac)
K073 CCACGCTGGCCGAGGACTGCGTGAAGACCAAG
K074 CTTGGTCTTCACGCAGTCCTCGGCCAGCGTGG

C191D (tgc → gac)
K075 CACGCTGGCCGAGTACGACGTGAAGACCAAG
K076 CTTGGTCTTCACGTCGTACTCGGCCAGCGTG

Y190D/C191D (tactgc → gacgac) altogether
K077 CACGCTGGCCGAGGACGACGTGAAGACCAAGG
K078 CCTTGGTCTTCACGTCGTCCTCGGCCAGCGTG

V183D/P184D (gtg → gat, ccc → gac) Stack V183 onto P184D
K079 GTGACGGCGTGAAGGATGACACCACGCTGGC
K080 GCCAGCGTGGTGTCATCCTTCACGCCGTCAC

P184D/Y190D (ccc → gac, tac → gac)
Stack Y190D onto P184D, Use Y190D primers K073 and K074

V181D/P184D/Y190D (gtg → gat, ccc → gac, tac → gac)
Stack V181D onto double P/Y mutant
K081 GGAGCGTGACGGCGATAAGGTGGACACCACG
K082 CGTGGTGTCCACCTTATCGCCGTCACGCTCC

L187D (ctg → gat)
K083 GAAGTGCCCACCACGGATGCCGAGTACTGC
K084 GCAGTACTCGGCATCCGTGGTGGGCACTTC

Y190A (tac → gcc)
K171 GCTGGCCGAGGCCTGCGTGAAGACCAAG
K172 CTTGGTCTTCACGCAGGCCTCGGCCAGC

Y190A/C191A (onto Y190A, tgc → gcc)
K175 CTGGCCGAGGCCGCCGTGAAGACCAAG
K176 CTTGGTCTTCACGGCGGCCTCGGCCAG

C93A (tgt → gcg)
K203 GAGACGGGGGACGTGGCGATCTCCATCCTC
K204 GAGGATGGAGATCGCCACGTCCCCCGTCTC

Amplification of hCdc34A proxmut gene fragment
K085 GGCGGCCATGGCTCGGCCGCTAGTG
K086 GGCGGCTCGAGTCAGGACTCCTCCGTG

ScUbiquitin mutagenesis

K48R (aag→ agg)
K089 CGTCTTATTTTCGCTGGTAGGCAGTTAGAAGATGGACG
K090 CGTCCATCTTCTAACTGCCTACCAGCGAAAATAAGACG

F45D (ttc → gac)
k380 GATCAACAACGTCTTATTGACGCTGGTAAGCAGTTAGAAG
k381 CTTCTAACTGCTTACCAGCGTCAATAAGACGTTGTTGATC

F45D onto K48R (ttc → gac)
k382 GATCAACAACGTCTTATTGACGCTGGTAGGCAGTTAGAAG
k383 CTTCTAACTGCCTACCAGCGTCAATAAGACGTTGTTGATC

A46D (gct → gat)
K207 CAACAACGTCTTATTTTCGATGGTAAGCAGTTAGAAGATG
K208 CATCTTCTAACTGCTTACCATCGAAAATAAGACGTTGTTG

A46D onto K48R (gct → gat)
k378 CAACAACGTCTTATTTTCGATGGTAGGCAGTTAGAAGATG
k379 CATCTTCTAACTGCCTACCATCGAAAATAAGACGTTGTTG

G47D (ggt → gat)
k125 CGTCTTATTTTTGCTGATAAGCAACTTGAAGATGGACG
k126 CGTCCATCTTCAAGTTGCTTATCAGCAAAAATAAGACG

G47D onto K48R (ggt → gat)
k141 CGTCTTATTTTTGCTGATAGGCAACTTGAAGATGGACG
k142 CGTCCATCTTCAAGTTGCCTATCAGCAAAAATAAGACG
