# Supplementary material for: Structural insights into E1 recognition and the ubiquitin-conjugating activity of the E2 enzyme Cdc34
Source: Nat Commun. 2019 Jul 24;10:3296. doi: 10.1038/s41467-019-11061-8 (PMC6656757; doi:10.1038/s41467-019-11061-8)
Supplement: Supplementary file 3 — Reporting Summary [file 41467_2019_11061_MOESM3_ESM.pdf]

nature research

Corresponding author(s): Shaun K. Olsen

Last updated by author(s): 5/7/2019

Reporting Summary

Nature Research wishes to improve the reproducibility of the work that we publish. This form provides structure for consistency and transparency in reporting. For further information on Nature Research policies, see Authors & Referees and the Editorial Policy Checklist.

Statistics

For all statistical analyses, confirm that the following items are present in the figure legend, table legend, main text, or Methods section.

n/a	Confirmed	
☐	☒	The exact sample size (n) for each experimental group/condition, given as a discrete number and unit of measurement
☐	☒	A statement on whether measurements were taken from distinct samples or whether the same sample was measured repeatedly
☐	☒	The statistical test(s) used AND whether they are one- or two-sided<br>*Only common tests should be described solely by name; describe more complex techniques in the Methods section.*
☐	☒	A description of all covariates tested
☐	☒	A description of any assumptions or corrections, such as tests of normality and adjustment for multiple comparisons
☐	☒	A full description of the statistical parameters including central tendency (e.g. means) or other basic estimates (e.g. regression coefficient) AND variation (e.g. standard deviation) or associated estimates of uncertainty (e.g. confidence intervals)
☒	☐	For null hypothesis testing, the test statistic (e.g. F, t, r) with confidence intervals, effect sizes, degrees of freedom and P value noted<br>*Give P values as exact values whenever suitable.*
☒	☐	For Bayesian analysis, information on the choice of priors and Markov chain Monte Carlo settings
☒	☐	For hierarchical and complex designs, identification of the appropriate level for tests and full reporting of outcomes
☒	☐	Estimates of effect sizes (e.g. Cohen's d, Pearson's r), indicating how they were calculated

Our web collection on statistics for biologists contains articles on many of the points above.

Software and code

Policy information about availability of computer code

Data collection	Crystallography data collection was conducted remotely using the in-house software of either NE-CAT or SER-CAT at the Advanced Photon Source.
Data analysis	Crystallography data analysis was conducted using the HKL2000, PHENIX, and COOT software suites.

For manuscripts utilizing custom algorithms or software that are central to the research but not yet described in published literature, software must be made available to editors/reviewers. We strongly encourage code deposition in a community repository (e.g. GitHub). See the Nature Research guidelines for submitting code & software for further information.

Data

Policy information about availability of data

All manuscripts must include a data availability statement. This statement should provide the following information, where applicable:

- Accession codes, unique identifiers, or web links for publicly available datasets
- A list of figures that have associated raw data
- A description of any restrictions on data availability

Atomic coordinates and structure factors are deposited in the RCSB with accession codes 6NYA, 6NYD, and 6NYO. The source data for all graphs, gel images, and blot images presented in these studies are provided as a Source Data file; any additional data may be requested from the corresponding author.

Field-specific reporting

Please select the one below that is the best fit for your research. If you are not sure, read the appropriate sections before making your selection.

☒ Life sciences ☐ Behavioural & social sciences ☐ Ecological, evolutionary & environmental sciences

For a reference copy of the document with all sections, see nature.com/documents/nr-reporting-summary-flat.pdf

Life sciences study design

All studies must disclose on these points even when the disclosure is negative.

Sample size	Due to the cleanliness of biochemical assays using purified proteins, these studies tend to have fairly robust differences in activity levels. We chose to do 4 independent technical replicates for each assay to allow for exclusion of one sample in the event of a technical error (i.e. gel ripping). If no technical error occurred, all four samples were included in the analysis. For mammalian cell studies, each experiment was conducted four independent times to ensure consistent results.
Data exclusions	Some replicates in the biochemical assays were excluded to due technical errors that resulted in unreadable or unreasonable results. If a technical error was identified prior to analysis, there was no cut-off for exclusion. If a technical error was not identified but a replicate was > 3 STD from the average of the other 3 replicates (pre-determined cut-off) it was excluded. Of note, only one replicate in the manuscript was excluded for being < 6, > 3 STD away from the average.
Replication	All of the biochemical data were initially produced in preliminary screening assays, then reproduced in publication quality replicates at a later time. All data were reproducible across these studies. The trends observed in mammalian studies were consistently produced four times, with no replication issues.
Randomization	We did not use experimental grouping in these studies, so no randomization was necessary.
Blinding	Initial biochemical data analyses for quantification were conducted using lane and construct numbers instead of mutant identification, however, the person conducting the quantification did purify the mutants and set up the assays, so due to memory it was not a fully-blind analysis. Since the method for quantification using ImageJ was essentially objective, we do not feel that blinding was necessary, and visual confirmation of the trends is available in the manuscript by providing gel images. For the mammalian studies, the method for semi-quantification of the protein bands to aid reader interpretation was not blinded, but was also not used to draw statistical conclusions. The mammalian flow cytometric data was not collected blind, but the gates were set per the WT data without influence from mutant datasets, and thus, should be unbiased.

Reporting for specific materials, systems and methods

We require information from authors about some types of materials, experimental systems and methods used in many studies. Here, indicate whether each material, system or method listed is relevant to your study. If you are not sure if a list item applies to your research, read the appropriate section before selecting a response.

Materials & experimental systems

n/a	Involved in the study	
☐	☒	Antibodies
☐	☒	Eukaryotic cell lines
☒	☐	Palaeontology
☒	☐	Animals and other organisms
☒	☐	Human research participants
☒	☐	Clinical data

Methods

n/a	Involved in the study	
☒	☐	ChIP-seq
☐	☒	Flow cytometry
☒	☐	MRI-based neuroimaging

Antibodies

Antibodies used	Mouse anti-Cdc34 antibody (Santa Cruz, cat. sc-28381) 1:1000 dilution; rabbit anti-p27 antibody (Cell Signaling, cat. 3686s) 1:1000 dilution; mouse anti-SKP1 antibody (BD Transduction Laboratories™, cat. 610530) 1:1000 dilution, rabbit anti-SKP2 antibody (Santa Cruz, cat. sc-7164) 1:1000 dilution, and mouse anti-β-actin antibody (Sigma, cat. A5316) 1:50,000 dilution.
Validation	The commercial antibodies have relative validation statement indicating they can be used for these species on the manufacturers' websites.

Eukaryotic cell lines

Policy information about cell lines

Cell line source(s)	U2OS and HEK293T cells were purchased from the American Type Culture Collection (ATCC).
Authentication	U2OS and HEK293T cells have the authentication information from ATCC.
Mycoplasma contamination	All cell lines were tested negative for mycoplasma contamination.
Commonly misidentified lines (See ICLAC register)	No such cell lines were used in this study.

Flow Cytometry

Plots

Confirm that:

☒ The axis labels state the marker and fluorochrome used (e.g. CD4-FITC).

☒ The axis scales are clearly visible. Include numbers along axes only for bottom left plot of group (a 'group' is an analysis of identical markers).

☐ All plots are contour plots with outliers or pseudocolor plots.

☒ A numerical value for number of cells or percentage (with statistics) is provided.

Methodology

Sample preparation	The flow cytometry was performed on U2OS cells, and the biological source of these cells is indicated above.<br>The procedure for cell cycle analysis: U2OS cells with control shRNA or Cdc34 shRNA as well as overexpression of Cdc34 WT or mutants were exposed to nocodazole synchronization for 16 hours and then released for 12 hours; meanwhile, asynchronized cells were used as a control population. Thereafter, cells were trypsinized and washed with cold PBS. Then, cells were stained with 10 µg/mL propidium iodide (PI) containing 100 µg/mL RNaseA before FACS analysis.
Instrument	BD FACSVerse™ flow cytometer
Software	The data were collected using BD FACSuite software, and the data were analyzed using FlowJo vX.0.7.
Cell population abundance	For cell cycle analysis, the single cell population abundance was about 75-80%; the cells were gated for single cell based on SSC +FSC in asynchronized group.
Gating strategy	For cell cycle analysis, the debris was excluded according to SSC+FSC gating and narrowed down to the single cell population. Thereafter, the DNA profile was optimized to adjust the G0/G1 peak to appear around channel 50 by changing the voltage.

☒ Tick this box to confirm that a figure exemplifying the gating strategy is provided in the Supplementary Information.
